# The Commonalities and Differences in Mitochondrial Dysfunction Between *ex vivo* and *in vivo* Myocardial Global Ischemia Rat Heart Models: Implications for Donation After Circulatory Death Research

**DOI:** 10.3389/fphys.2020.00681

**Published:** 2020-06-23

**Authors:** Mohammed Quader, Oluwatoyin Akande, Stefano Toldo, Renee Cholyway, Le Kang, Edward J. Lesnefsky, Qun Chen

**Affiliations:** ^1^Hunter Holmes McGuire Veterans Administration Medical Center, Richmond, VA, United States; ^2^Division of Thoracic and Cardiovascular Surgery, Department of Surgery, Virginia Commonwealth University, Richmond, VA, United States; ^3^Pauley Heart Center, Virginia Commonwealth University, Richmond, VA, United States; ^4^Division of Cardiology, Department of Internal Medicine, Virginia Commonwealth University, Richmond, VA, United States; ^5^Department of Biostatistics, Virginia Commonwealth University, Richmond, VA, United States

**Keywords:** circulatory death donors, brain death donors, ischemia, heart transplantation, mitochondria

## Abstract

Heart transplantation is the ultimate treatment option for patients with advanced heart failure. Since hearts from donation after brain death (DBD) donors are limited, donation after circulatory death (DCD) donor hearts could be another source for heart transplantation. DCD process involves ischemia-reperfusion (IR) injury. Mitochondrial dysfunction contributes to IR and is well established in the *ex vivo* (buffer perfused) ischemia animal model. However, DCD hearts undergo *in vivo* ischemia with a variable “ischemic period.” In addition, the DCD hearts are exposed to an intense catecholamine surge that is not seen with *ex vivo* perfused hearts. Thus, the severity of mitochondrial damage in *in vivo* ischemia hearts could differ from the *ex vivo* ischemia hearts even following the same period of ischemia. The aim of our current study is to identify the mitochondrial dysfunction in DCD hearts and propose strategies to protect mitochondria. Adult Sprague Dawley rat hearts underwent *in vivo* or *ex vivo* ischemia for 25 min. Subsarcolemmal mitochondria (SSM) and interfibrillar mitochondria (IFM) were isolated from hearts following ischemia. We found that both *ex vivo* and *in vivo* ischemia led to decreased oxidative phosphorylation in SSM and IFM compared to time control or DBD hearts. The proportion of damage to SSM and IFM, including proton leak through the inner membrane, was higher with *ex vivo* ischemia compare to *in vivo* ischemia. Time control hearts showed a decrease in SSM and IFM function compared to DBD hearts. The calcium retention capacity (CRC) was also decreased in SSM and IFM with *ex vivo* and *in vivo* ischemia, indicating that ischemic damage to mitochondria sensitizes mitochondrial permeability transition pores (MPTP). Our study found differential mitochondrial damage between the *in vivo* ischemia and the *ex vivo* ischemia setup. Therefore, consideration should be given to the mode of ischemia while evaluating and testing myocardial protective interventions targeting mitochondria to reduce IR injury in hearts.

## Introduction

Heart transplantation is the ultimate treatment option for patients with advanced heart failure; however, this option is limited due to the available donor hearts, which primarily come from donation after brain death (DBD) donors (Colvin et al., 2018). Donation after circulatory death (DCD) donors can potentially expand the donor heart pool (NHS Blood and Transplant, n.d.). Since the DCD process involves ischemia-reperfusion (IR) injury, hearts from such donors are not routinely utilized for transplantation. Therefore, myocardial protective interventions specifically targeted at tempering IR injury and protecting remaining viable myocardium is essential for DCD hearts to be considered for transplantation.

Mitochondria are integral to the functioning of myocytes, which are one of the most metabolically active cells in the body. While mitochondria are essential to maintain cardiac function, dysfunctional mitochondria are also the sources of cardiac injury during IR (Lesnefsky et al., 2016, 2017). The dedicated work of researchers has led to understanding the mechanisms involved in damage to the mitochondria from IR injury (Gustafsson and Gottlieb, 2008; Murphy and Steenbergen, 2008; Lesnefsky et al., 2016, 2017). Depending on the duration of the ischemic period, the mitochondrial function can be impaired transiently or damaged, permanently leading to cell death. Principal sites of damage to mitochondria are in the electron transport chain (ETC) (Lesnefsky et al., 1997; Chen et al., 2006a). The impaired ETC increases cardiac injury during IR by reducing energy production, increasing reactive oxygen species (ROS) generation, and sensitizing mitochondrial permeability transition pore (MPTP) opening (Gustafsson and Gottlieb, 2008; Murphy and Steenbergen, 2008; Lesnefsky et al., 2016, 2017). MPTP opening increases the permeability of the inner mitochondrial membrane that induces mitochondrial matrix swelling leading to the rupture of the outer mitochondrial membrane. Mitochondrial proteins, including cytochrome *c* and apoptosis-inducing factor (AIF), are released into cytosol leading to apoptosis in caspase-dependent and caspase-independent manner (Green and Reed, 1998; Yu et al., 2002; Chen et al., 2011). Thus, the protection of mitochondria is a promising strategy to decrease cardiac injury during IR in DCD hearts.

Since IR is a natural event and cannot be avoided in the DCD process, it is critical to identify mitochondrial dysfunction and develop proper strategies to protect them. Currently, a vast amount of knowledge gained on the mitochondrial dysfunction in the setting of IR is based on *ex vivo* (buffer perfused) ischemia model (Gustafsson and Gottlieb, 2008; Lesnefsky et al., 2016, 2017). The advantage of the *ex vivo* model is that the onset of ischemia and “ischemic period” can be precisely set when the perfusion flow is stopped. However, the “ischemic period” in a DCD heart, henceforth referred to as *in vivo* ischemia, varies according to the cardiorespiratory reserve of the donor (Iyer et al., 2016; White et al., 2016). The IR injury in the *in vivo* ischemic heart is unique in that the onset of ischemia is not abrupt, the ischemic myocyte is stretched from the distention of heart chambers, and that there is a variable duration of arrhythmia prior to asystole. In addition, an intense catecholamine surge occurs in the *in vivo* ischemia setup that is not seen with the *ex vivo* ischemia model (Iyer et al., 2016; White et al., 2016). Thus, the severity of mitochondrial damage in *in vivo* ischemia hearts could differ from the *ex vivo* ischemia hearts for the same duration of ischemia.

There are two populations of cardiac mitochondria: subsarcolemmal mitochondria (SSM) located underneath the plasma membrane and interfibrillar mitochondria (IFM) that exist between the myofibrils (Palmer et al., 1977; Lesnefsky et al., 2001b). In the *ex vivo* ischemia model, IFM are known to be more resistant to ischemic damage compared to SSM, especially in larger animal models (Lesnefsky et al., 1997). An IFM defect is more pronounced in aging, heart failure, and diabetic hearts. However, it is unclear if the *in vivo* ischemia setup also shows less damage to IFM. The aim of our study is to explore the commonalities and differences in mitochondrial dysfunction as observed with *in vivo ischemia* (DCD) or *ex vivo* (buffer perfused) ischemia models. Knowledge gained from such a study will lay the foundation for future interventions aimed at protecting DCD hearts and preserving their function.

## Materials and Methods

### Ethical Aspects

All experimental animals were cared in accordance with institutional guidelines and the *Guide for the Care and Use of Laboratory Animals*, published by the National Institutes of Health (NIH Publication No. 86-23, revised 2011) (N. R. Council, 2011). The study protocols were approved by the Hunter Holmes McGuire Veterans Administration Hospital and Virginia Commonwealth University’s Institutional Animal Care and Use Committee.

### *In vivo* and *ex vivo* Experimental Animal Models

**FIGURE 1:**
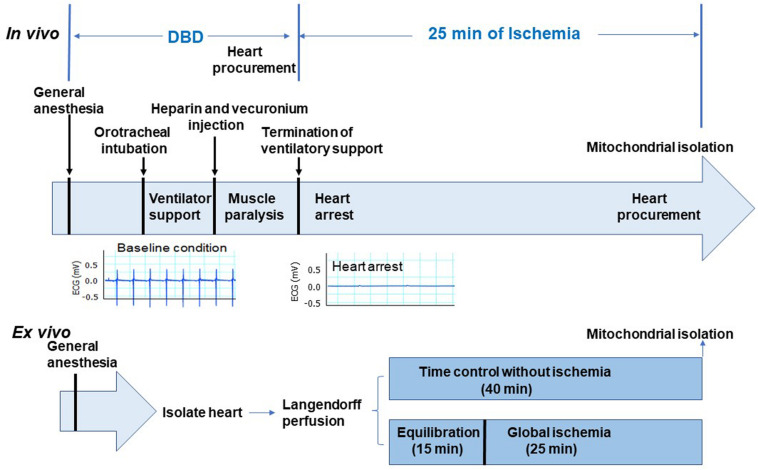
Depiction of the experimental protocol. The upper panel shows the *in vivo* ischemia experimental protocol. Sprague Dawley male rats were anesthetized with pentobarbital and connected to the ventilator through an endotracheal tube. Heparin (300 U) was administered intraperitoneally. Vecuronium was used to induce muscle paralysis via intramuscular injection. EKG monitor was established after anesthesia. Hearts in the donation after brain death (DBD) group were collected without stopping ventilation. In the *in vivo* ischemia group, the ventilation was stopped 5 min after vecuronium injection. EKG was continuously monitored. Asystole was observed between 9 and 12 min after apnea. Interestingly, ventricular fibrillation was rare in *in vivo* ischemia hearts (see representative EKG panel). After 25 min of ischemia, hearts were procured for mitochondrial isolation. Lower panel shows the *ex vivo* experimental protocol. The isolated hearts were mounted in the Langendorff set-up with Krebs–Henseleit buffer perfusion. After 15 min of equilibration perfusion, the isolated hearts underwent 25 min of global ischemia at 37°C. In the time control group, hearts were buffer-perfused without ischemia. Hearts were collected for mitochondrial isolation at the end of 25 min ischemia time in *ex vivo* group and after 40 min of perfusion in the time control group.

*In vivo* global ischemia model adult male Sprague Dawley rats (10–15 weeks old, weighing <400 g) were randomly assigned to a DBD (*n* = 6) or an *in vivo ischemia* group (*n* = 5). The rats were placed on a heating pad to maintain the body temperature of 37°C, anesthetized using sodium pentobarbital (100 mg/kg) administered intraperitoneally, followed by endotracheal intubation with 14-gauge angio-cath for respiratory support. Heart activity was monitored by continuous EKG recording. Heparin (300 U) was administered intraperitoneally and allowed to circulate for 1 min, followed by intramuscular administration of a paralytic agent (vecuronium bromide, 40 mg/kg) and allowed to circulate for 5 min. Rats in the DBD group underwent a thoracotomy, and hearts were procured while beating. Hearts were then processed for mitochondrial isolation, as described previously (Chen et al., 2006a). In the *in vivo* ischemia group, the ventilator support was terminated, leading to instantaneous respiratory arrest followed by cardiac asystole as monitored by the continuous EKG. Twenty-five minutes following termination of the ventilator, thoracotomy was performed, hearts procured and processed for mitochondrial isolation. Figure 1 shows a schematic of procedures and timing for the DBD and *in vivo* ischemia hearts.

*Ex vivo* global ischemia setup – the rats were anesthetized using sodium pentobarbital (100 mg/kg) administered intraperitoneally. Hearts were then isolated under general anesthesia and mounted on the Langendorff setup (Chen et al., 2006a). Hearts were perfused at a constant pressure of 72 mmHg with a modified Krebs–Henseleit (K–H) buffer (115 mM NaCl, 4.0 mM KCl, 2.5 mM CaCl_2_, 26 mM NaHCO_3_, 1.1 mM MgSO_4_, 0.9 mM KH_2_PO_4_, 5.5 mM glucose, and 5 IU of insulin/liter), oxygenated with 95% O_2_ – 5% CO_2_ to maintain a pH of 7.4. Rat hearts were assigned to time control (*n* = 11) or *ex vivo* ischemia (*n* = 9) groups. In the *ex vivo* ischemia group, hearts were perfused for 15 min equilibration with K-H buffer followed by 25 min of global ischemia at 37°C. In the time control group, hearts were only buffer perfused for 40 min without ischemia (Figure 1, lower panel). The hearts were then procured for mitochondrial isolation at the end of the perfusion.

### Isolation of Rat Heart Mitochondria

The DBD and *in vivo* ischemia hearts once procured fresh were placed into cold (4°C) buffer A [100 mM KCl, 50 mM 3-(*N*-morpholino) propanesulfonic acid (MOPS), 1 mM ethylene glycol-bis(β-aminoethyl ether)-N,N,N,N’-tetraacetic acid (EGTA), 5 mM MgSO4.7 H_2_O, and 1 mM ATP, pH 7.4]. Mitochondria were isolated using our previously published protocol (Chen et al., 2006a). Briefly, the minced cardiac tissue was placed in buffer A containing 0.2% bovine serum albumin and homogenized with a polytron tissue processor (Brinkmann Instruments, Westbury, NY, United States) for 2.5 s at the 10,000 rpm. The polytron homogenate was centrifuged at 500 × *g* for 10 min. The supernatant was further centrifuged at 3,000×*g* for 10 min to sediment SSM. The pellet was re-suspended in buffer A and homogenized and incubated with trypsin (5 mg/g wet weight) for 10 min at 4°C. The homogenate was centrifuged at 500 × *g* for 10 min, and the supernatant was further centrifuged at 3,000 × *g* for 10 min to sediment IFM. The SSM and IFM were washed twice and then suspended in 100 mM KCl, 50 mM MOPS, and 0.5 mM EGTA. Mitochondrial protein content was measured by the Lowry method (Chen et al., 2006a), using bovine serum album as a standard.

### Mitochondrial Oxidative Phosphorylation

Oxygen consumption in mitochondria was measured using a Clark-type oxygen electrode (Strathkelvin Instruments, North Lanarkshire, United Kingdom) at 30°C, as previously described (Lesnefsky et al., 1997). Mitochondria were incubated in 80 mM KCl, 50 mM MOPS, 1 mM (EGTA, 5 mM KH_2_PO_4_, and 1 mg defatted, dialyzed bovine serum albumin/mL at pH 7.4. Glutamate (20 mM, complex I substrate), succinate (20 mM) plus 7.5 μM rotenone (complex II substrate), and N,N,N′,N′ tetramethyl p-phenylenediamine, 1 mM (TPMD) ascorbate (10 mM, complex IV substrate) + 7.5 μM rotenone were used (Chen et al., 2006b).

### Measurement of Respiratory Enzyme Activity

Frozen and thawed SSM/IFM were solubilized in 5% cholate (pH 7.2), then diluted with KME (100 mM KCl, 50 mM MOPS, and 1 mM EGTA) buffer to 0.1% protein concentration. Hewlett-Packard model 8453 spectrophotometer (Waldbronn, Germany) was used to measure respiratory enzyme activities. Complex II activity was measured as the rate of oxidation of dichlorophenol-indophenol (DCPIP) at 600 nm in the presence of mitochondria (2 μg/mL), 1 mM oxidized decylubiquinone, and 1 M succinate. Complex II activity was expressed as the thenoyltrifluoroacetone (TTFA) sensitive rate (complex II activity without TTFA subtracted complex II activity with TTFA). Complex III activity was measured as the initial rate of the reduction of cytochrome *c* at 550 nm on the addition of 40 μM reduced decylubiquinone and 1 mM cytochrome *c* to 3 μg/mL mitochondrial protein with or without 100 μM antimycin A. Complex III activity was expressed as antimycin A sensitive rate (complex III activity without antimycin A subtracted complex III activity with antimycin A). Citrate synthase (CS) activity was quantified in cholate-solubilized mitochondria by measuring the rate of 5,5-dithiobis(nitrobenzoic acid)-reactive reduced coenzyme A (412 nm, 13,600 M/cm) at 37°C as previously described (Lesnefsky et al., 1997).

### Measurement of Cytochrome Content

Mitochondrial cytochrome contents (c, b, c1, and aa3) were determined using our published method (Lesnefsky et al., 1997). SSM/IFM (0.4 mg) were first solubilized in 2% DOC and then resuspended in 10 mM sodium phosphate, pH 7.0. Cytochrome contents were determined by the difference between oxidized and reduced spectra. Sodium dithionite was used to generate spectra (Lesnefsky et al., 1997; Chen et al., 2003).

### Calcium Retention Capacity in Isolated Mitochondria

Calcium retention capacity (CRC) was used to assess calcium-induced MPTP opening in isolated mitochondria using LS-5 fluorimeter (Perkin Elmer, Waltham, MA, United States) (Paillard et al., 2009). Mitochondria (250 μg) were incubated in buffer containing 150 mM sucrose, 50 mM KCl, 2 mM KH_2_PO_4_, 5 mM succinate in 20 mM Tris/HCl, pH 7.4. MPTP was induced by sequential pulses of calcium (5 nmol/min). Succinate was used as a substrate in that the CRC is much higher in mitochondria oxidizing a complex II substrate compared to a complex I substrate (Madungwe et al., 2016; Matsuura et al., 2017). Extra-mitochondrial Ca^2+^ concentration was recorded with 0.5 μM Calcium Green-5N, and fluorescence monitored with excitation and emission wavelengths set at 500 and 530 nm, respectively.

### Measurement of H_2_O_2_ Generation

The rate of H_2_O_2_ production in freshly isolated SSM/IFM was determined using the oxidation of the fluorogenic indicator amplex red in the presence of horseradish peroxidase. The concentrations of horseradish peroxidase and amplex red in the incubation were 0.1 U/mL and 50 μM, respectively. Fluorescence was recorded in a fluorimeter (LC5, Perkin Elmer Life Sciences) with 530 nm excitation and 590 nm emission wavelengths. Standard curves obtained by adding known amounts of H_2_O_2_ to assay medium in the presence of the reactants (amplex red and horseradish peroxidase) were linear up to 2 μM. In a typical experiment, SSM/IFM were incubated at 0.1 mg of protein/mL at 30°C. H_2_O_2_ production was initiated in mitochondria using glutamate (20 mM) or succinate (20 mM) + rotenone (7.5 μM) as complex I and complex II substrates, respectively (Chen et al., 2003).

### Statistical Analysis

**FIGURE 2:**
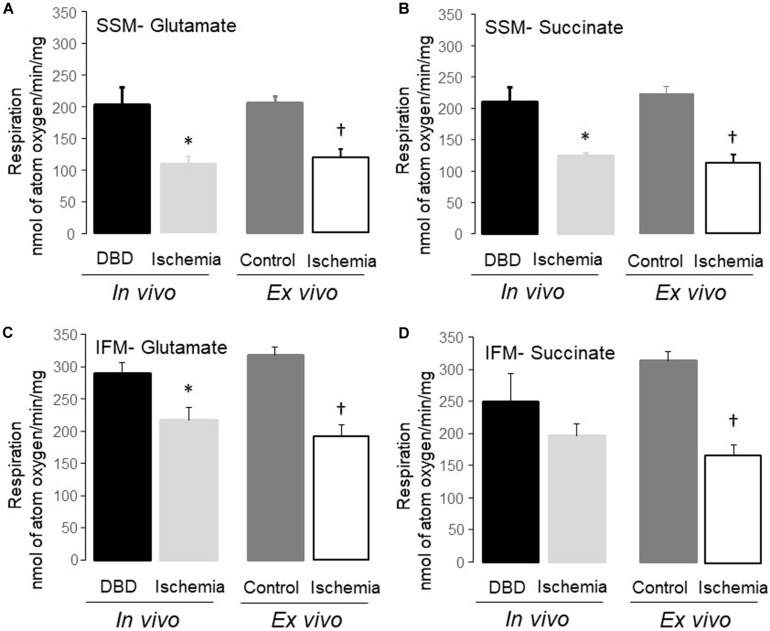
Uncoupled respiration was decreased in subsarcolemmal mitochondria (SSM) and interfibrillar mitochondria (IFM) following *in vivo* and *ex vivo* ischemia. Compared to donation after brain death (DBD) hearts, 25 min of *in vivo* ischemia led to decreased rates of respiration in SSM using glutamate and succinate as complex I **(A)**, and complex II **(B)** substrates, respectively. Similar to the *in vivo* ischemia study, *ex vivo* ischemia also led to decreased uncoupled respiration in SSM oxidizing complex I and II substrates **(A,B)** compared to time control. *In vivo* ischemia led to decreased respiration in IFM using complex I substrates **(C)**. Succinate oxidation was not altered in IFM from *in vivo* ischemia hearts **(D)**. *Ex vivo* ischemia led to decreased oxidative phosphorylation in IFM oxidizing complex I and II substrates **(C,D)** compared to time control. Data are expressed as mean ± SEM; *p < 0.05 vs DBD; ^†^*p* < 0.05 vs time control. Number of rat hearts for each group are, DBD = 6, *in vivo* ischemia = 5, time control = 11, and *ex vivo* ischemia = 9. nAO, nanomole of atom oxygen. Statistical method: one-way ANOVA using the Student–Newman–Keuls analysis for groups comparisons with an individual comparison *p* < 0.05 considered significant.

Continuous variables were expressed as a mean ± standard error of the mean, or a median with interquartile range (IQR) if appropriate. Data were tested for normality and equal variance before performing statistical comparisons between four groups (DBD, *in vivo* ischemia, *ex vivo* time control, and *ex vivo* ischemia) based on parametric one-way analysis of variance (ANOVA) (Steel and Torrie, 1960). Alternatively, if the parametric one-way ANOVA model assumption was violated, the non-parametric Kruskal–Wallis one-way ANOVA was considered. A *p*-value less than 0.05 from either parametric or non-parametric one-way ANOVA was considered significant to reject the overall null hypothesis that data shares the same underlying distribution across four groups. If the overall ANOVA was significant, pairwise group comparisons were further conducted using the Student–Newman–Keuls analysis, or Dunn’s test if appropriate, with necessary multiplicity adjustments (Steel and Torrie, 1960). Specifically, comparisons among the four groups (DBD, *in vivo* ischemia, *ex vivo* time control, and *ex vivo* ischemia) for SSM with succinate as substrate for state 3 respiration, 2 mM ADP-stimulated respiration and ADP/O ratio; SSM TMPD-ascorbate oxidation, and IFM respiratory control ratio (RCR) with glutamate as substrate failed the test of normality and were compared using the Kruskal–Wallis one-way ANOVA followed by Dunn’s test for multiple groups. Data in Tables 1, 2 and Figure 2 were analyzed using this approach. Comparisons between two groups (DBD and *in vivo* ischemia in Tables 3, 4) were performed using a non-paired Student *t*-test (two-tails) after data passed tests of normality. Comparison of SSM cytochrome *c* content between DBD and *in vivo* ischemia groups in Table 4 was performed with the Mann–Whitney rank-sum test as the data failed the test of normality. Outlier data was defined based upon the difference between the individual data point and the mean of the remaining data greater than twice the standard deviation. Based upon this criteria, three measurements were identified as outlier data and were excluded before statistical comparisons were performed (*in vivo* ischemia group: one determination of SSM state 4 respiration with glutamate as substrate, Table 1 and one measurement of IFM cytochrome *aa*_3_ content, Table 4; DBD group: one measurement of IFM state 3 respiration with succinate as substrate, Table 2). SigmaStat software (version 3.5, Systat Software, Inc., San Jose, CA, United States) and R (version 4.0.0, R Foundation for Statistical Computing, Vienna, Austria) were used for the statistical analysis.

**TABLE 1:** Comparison of SSM oxidative phosphorylation between *in vivo* and *ex vivo* ischemia heart models.

**SSM**	***In vivo* model**		***Ex vivo* model**	
	**DBD (*n* = 6)**	***In vivo* ischemia (*n* = 5)**	**Time control (*n* = 11)**	***Ex vivo* ischemia (*n* = 9)**
Mitochondrial protein yield (mg/g tissue)	15.8 ± 1.2	12.4 ± 0.7*	10.1 ± 0.7*	9.0 ± 0.8*^,‡^
OXPHOS	With glutamate			
State 3 (nAO/mg/min)	198 ± 15	134 ± 8*	192 ± 6^‡^	111 ± 10*^,^^†^
State 4 (nAO/mg/min)	18 ± 2	25 ± 4	27 ± 3*	58 ± 5*^,†,‡^
RCR	13.5 ± 1.8	5.6 ± 1.6*	8.2 ± 1.0*	2.0 ± 0.2*^,†,‡^
ADP/O	3.15 ± 0.10	2.70 ± 0.07	3.05 ± 0.12	2.75 ± 0.09
2mM ADP (nAO/mg/min)	205 ± 20	121 ± 11*	201 ± 10^‡^	117 ± 11*^,†^
OXPHOS	With succinate			
State 3 (nAO/mg/min)	247 (68)	141 (16)*	236 (54)^‡^	98 (56)*^,†,‡^
State 4 (nAO/mg/min)	78 ± 10	61 ± 5	83 ± 9	67 ± 5
RCR	3.5 ± 0.5	2.3 ± 0.3*	3.0 ± 0.2*,^‡^	1.6 ± 0.1*^,†,‡^
ADP/O	1.62 (0.13)	1.60 (0.03)	1.67 (0.16)	1.52 (0.32)
2 mM ADP (nAO/mg/min)	243 (50)	131 (16)*	228 (53)^‡^	108 (64)*^,†^
OXPHOS	With TMPD-ascorbate			
2 mM ADP (nAO/mg/min)	633 (32)	441 (14)*	671 (92)	393 (79)*^,†^

SSM, subsarcolemmal mitochondria; DBD, donation after brain death; OXPHOS, oxidative phosphorylation; nAO, nanomole of atom oxygen; ADP, adenosine diphosphate; RCR, respiratory control ratio; TMPD, N,N,N,N’-tetramethyl-p-phenylenediamine; mean ± SEM or median (IQR). *p < 0.05 vs DBD; ^†^*p* < 0.05 vs time control; ^‡^*p* < 0.05 vs *in vivo* ischemia. Statistical methods as described in detail in section “Materials and Methods.” Parametric one-way ANOVA with the Student–Newman–Keuls analysis for pairwise group comparisons was used when data passed normality and equal variance tests which includes all comparisons except the four noted next. Comparisons of state 3 respiration, 2 mM ADP-stimulated respiration, and ADP/O ratio with succinate as substrate and respiration with TMPD-ascorbate as substrate were compared using non-parametric Kruskal–Wallis one-way analysis of variance (ANOVA) on ranks followed by Dunn’s analysis for individual comparisons due to data failing the test of normality and equal variance These data were expressed as median (IQR) accordingly.

**TABLE 2:** Comparison of IFM oxidative phosphorylation between *in vivo and ex vivo* ischemia heart models.

**IFM**	***In vivo* model**		***Ex vivo* model**	
	**DBD (*n* = 6)**	***In vivo* ischemia (*n* = 5)**	**Time control (*n* = 11)**	***Ex vivo* ischemia (*n* = 9)**
Mitochondrial protein yield (mg/g tissue)	14.5 ± 0.7	12.6 ± 1.4	10.2 ± 0.9*	8.3 ± 0.5*^,^^‡^
OXPHOS	With glutamate			
State 3 (nAO/mg/min)	261 ± 14	221 ± 20	277 ± 11^‡^	175 ± 14*^,^^†^^,‡^
State 4 (nAO/mg/min)	25 ± 2	39 ± 5*	33 ± 3	67 ± 3*^,^^†^^,‡^
RCR	11.5 (1.0)	5.0 (2.0)*	8.6 (2.9)^‡^	2.3 (0.8)*^,^^†^^,‡^
ADP/O	3.25 ± 0.07	2.87 ± 0.05	3.13 ± 0.10	2.85 ± 0.11
2 mM ADP (nAO/mg/min)	275 ± 17	226 ± 22*	309 ± 10^‡^	183 ± 14*^,^^†^^,‡^
OXPHOS	With Succinate			
State 3 (nAO/mg/min)	294 ± 24	228 ± 15	241 ± 14	163 ± 13^†,‡^
State 4 (nAO/mg/min)	102 ± 20	89 ± 4	83 ± 9	98 ± 7
RCR	3.6 ± 0.4	2.6 ± 0.2*	3.0 ± 0.2	1.7 ± 0.1^†,‡^
ADP/O	1.51 ± 0.13	1.71 ± 0.10	1.66 ± 0.08	1.61 ± 0.16
2mM ADP (nAO/mg/min)	302 ± 57	211 ± 17*	337 ± 17^‡^	184 ± 18*^,^^†^
OXPHOS	With TMPD-ascorbate			
2 mM ADP (nAO/mg/min)	733 ± 69	417 ± 87*	991 ± 30*	563 ± 46*^,^^†^

IFM, interfibrillar mitochondria; DBD, donation after brain death; OXPHOS, oxidative phosphorylation; nAO, nanomole of atom oxygen; ADP, adenosine diphosphate; RCR, respiratory control ratio; OXPHOS, oxidative phosphorylation; TMPD, N,N,N’,N’-tetramethyl-p-phenylenediamine; mean ± SEM or median (IQR). *p < 0.05 vs DBD; ^†^*p* < 0.05 vs time control; ^‡^*p* < 0.05 vs *in vivo ischemia*. Statistical methods as described in detail in section “Materials and Methods.” Parametric one-way ANOVA with the Student–Newman–Keuls analysis for pairwise group comparisons was used when data passed normality and equal variance tests, which includes all comparisons except the following. The comparison of RCR with glutamate as substrate was compared using non-parametric Kruskal–Wallis one-way analysis of variance (ANOVA) on ranks followed by Dunn’s analysis for individual comparisons due to data failing the test of normality and equal variance. The data were expressed as median (IQR) accordingly.

**TABLE 3:** Respiratory enzyme activity in SSM and IFM from DBD and *in vivo* ischemia hearts.

	**SSM-DBD (*n* = 6)**	**SSM-*in vivo* Isc (*n* = 5)**	**IFM-DBD (*n* = 6)**	**IFM-*in vivo* Isc (*n* = 5)**
Complex II (mU/mg)	374 ± 49	300 ± 63	391 ± 62	393 ± 33
Complex III (mU/mg)	4,743 ± 274	3,831 ± 982	4,264 ± 684	2,650 ± 222
Citrate synthase (mU/mg)	1,609 ± 134	1,622 ± 353	2,586 ± 365	3,066 ± 302

*ETC, electron transport chain; SSM, subsarcolemmal mitochondria; IFM, interfibrillar mitochondria; DBD, donation after brain death; *in vivo* Isc, *in vivo* ischemia; mean ± SEM. *p* = NS. Statistical method: two tail non-paired Student *t*-test.*

**TABLE 4:** Cytochrome content in SSM and IFM from DBD vs *in vivo* ischemia hearts.

	**SSM-DBD (*n* = 5)**	**SSM-*in vivo* Isc (*n* = 5)**	**IFM-DBD (*n* = 5)**	**IFM-*in vivo* Isc (*n* = 5)**
c (nmol/mg)	0.191 (0.081)	0.138 (0.022)*	0.313 ± 0.018	0.200 ± 0.023^†^
b (nmol/mg)	0.209 ± 0.024	0.170 ± 0.012	0.265 ± 0.021	0.240 ± 0.003
c1 (nmol/mg)	0.121 ± 0.015	0.140 ± 0.018	0.136 ± 0.013	0.090 ± 0.020
aa3 (nmol/mg)	0.339 ± 0.067	0.200 ± 0.057	0.591 ± 0.038	0.310 ± 0.068^†^

SSM, subsarcolemmal mitochondria; IFM, interfibrillar mitochondria; DBD, donation after brain death; *in vivo* Isc, *in vivo* ischemia; mean ± SEM or median (IQR) if appropriate. *p < 0.05 vs DBD-SSM. ^†^*p* < 0.05 vs DBD-IFM. Statistical method: two tail non-paired Student *t*-test. The non-parametric Mann–Whitney rank sum test was used to compare SSM cytochrome *c* content between DBD and *in vivo* ischemia groups due to failure of the data to meet tests of normality and equal variance. The data were expressed as median (IQR) accordingly.

## Results

### Injury to ETC in SSM Is Seen With Both *in vivo* and *ex vivo* Ischemia but in Varying Amounts – Table 1

Mitochondrial protein yield, reflective of physically intact mitochondria, was decreased in SSM from *in vivo* ischemia, *ex vivo* ischemia, and time control groups compared to the DBD hearts. In particular, the protein yield in the *ex vivo* ischemia group was significantly less compared to the *in vivo* ischemia group. Compared to DBD hearts, the rate of state 3 respiration (ADP-stimulated) in SSM oxidizing complex I substrate (glutamate) was significantly decreased by 33 and 44% for *in vivo* and *ex vivo* ischemia groups, respectively. Compared to the DBD group, state 4 respiration (ADP-limited) in SSM was not significantly different in *in vivo* ischemia group. Interestingly, time control perfusion alone led to a significant increase in state 4 respiration in SSM compared to DBD hearts. *Ex vivo* ischemia significantly increased state 4 respiration in SSM compared to *in vivo* ischemia group. RCR was calculated as a ratio of state 3/state 4 respiration. Compared to DBD SSM, all other groups had significantly reduced RCR (Table 1). The RCR was significantly lower in the SSM of *ex vivo* ischemia group compared to the *in vivo* ischemia group due to both decreased state 3 respiration and increased state 4 respiration. The maximal rate of ADP-stimulated respiration using a saturating concentration of ADP (2 mM) was equally decreased in *in vivo* ischemia and *ex vivo* ischemia groups compared to DBD or time control groups. ADP-stimulated OXPHOS was also decreased in *in vivo* ischemia SSM oxidizing complex II substrate (succinate) in a manner similar to what is observed with complex I substrate (Table 1). The RCR, in particular, were significantly less in SSM exposed to *ex vivo* ischemia compared to *in vivo* ischemia, both with complex I and II substrates. A decrease in OXPHOS can be due to the impaired ETC or from damage to complex V that limits phosphorylation. To localize the site of the defect to the ETC and to exclude the phosphorylation apparatus, a chemical uncoupling agent dinitrophenol (DNP) was used. DNP transfers proton across the inner membrane collapsing the inner membrane potential and essentially bypassing complex V (Lesnefsky et al., 1997). DNP stimulated respiration was decreased in both *in vivo* ischemia and *ex vivo* ischemia SSM oxidizing complex I (Figure 2A) and II (Figure 2B) substrates. This finding localizes the defect in OXPHOS to the ETC rather than the complex V and the phosphorylation apparatus. Interestingly, the higher amounts of damage to SSM as seen with *ex vivo* ischemia compared to *in vivo* ischemia for state 3 and 4 respiration was not seen with DNP stimulated respiration.

### Damage to IFM Was Higher With *ex vivo* Ischemia Compared to *in vivo* Ischemia – Table 2

There was no significant difference in the protein yield from IFM of *in vivo ischemia* and DBD groups; however, there was a significant decrease in protein yield from IFM of *ex vivo* ischemia and buffer-perfused hearts compared to DBD hearts (Table 2). In particular, the protein yield from IFM in *ex vivo* ischemia group was significantly less compared to *in vivo* ischemia group (Table 2). Compared to DBD hearts, the rate of state 3 respiration was not significantly decreased in *in vivo* ischemia IFM oxidizing complex I substrate. However, the state 4 respiration was increased in *in vivo* ischemia IFM compared to DBD. This led to significantly decreased RCR in *in vivo* ischemia IFM compared to IFM from DBD hearts (Table 2). *Ex vivo* ischemia led to decreased state 3 respiration and increased state 4 respiration in IFM using complex I substrate compared to time control IFM (Table 2). In IFM state 3 respiration oxidizing complex II substrate was numerically less in *in vivo* ischemia group compared to DBD, but was not statistically significant. The state 3 respiration in IFM utilizing complex II substrate was significantly less in *ex vivo* ischemia group compared to time control. The *ex vivo* ischemia also led to a significantly decreased state 3 respiration and RCR in IFM oxidizing complex II substrate compared to *in vivo* ischemia (Table 2). The state 3 respiration was significantly lower in IFM following *ex vivo* ischemia compared to *in vivo* ischemia for the oxidation of both complex I and complex II substrates, suggesting the more severe damage happening in *ex vivo* ischemia IFM. The saturated ADP (2 mM) stimulated respiration was also decreased in IFM with *ex vivo* ischemia compared to *in vivo* ischemia for the oxidation of complex I substrate but not with complex II substrate.

Dinitrophenol-stimulated respiration was also significantly decreased in *in vivo* ischemia IFM oxidizing complex I (Figure 2C) substrates but not complex II substrate compared to DBD IFM. DNP-stimulated respiration was uniformly decreased in IFM from *ex vivo* ischemia hearts oxidizing complex I (Figure 2C), II (Figure 2D) substrates compared to time control IFM. The degree of decrease in DNP-stimulated OXPHOS in IFM of the *in vivo ischemia* group was smaller compared to the *ex vivo* ischemia group.

### *In vivo* Ischemia Led to Decreased Respiratory Enzyme Activity – Table 3

The activity of complex II and III in SSM and IFM from *in vivo* ischemia hearts was not altered compared to DBD hearts (Table 3). CS (a matrix enzyme) activity was also not altered in *in vivo* heart mitochondria compared to DBD hearts.

### *In vivo* Ischemia Led to Decreased Cytochrome *c* Content in Both SSM and IFM – Table 4

Mitochondrial cytochrome *c* content was decreased in both SSM (34% less) and IFM (35% less) following *in vivo* ischemia compared to DBD control hearts. The *in vivo* ischemia did not alter the contents of aa3 (an index of cytochrome oxidase protein content). b and c1 (components of complex III) in SSM compared to DBD hearts. There were no differences in the contents of cytochrome b and c1 in IFM between DBD and *in vivo* ischemia hearts. The *in vivo* ischemia led to decreased aa3 content in IFM compared to DBD hearts.

### Both *in vivo* Ischemia and *ex vivo* Ischemia Sensitized MPTP Opening – Figure 3

**FIGURE 3:**
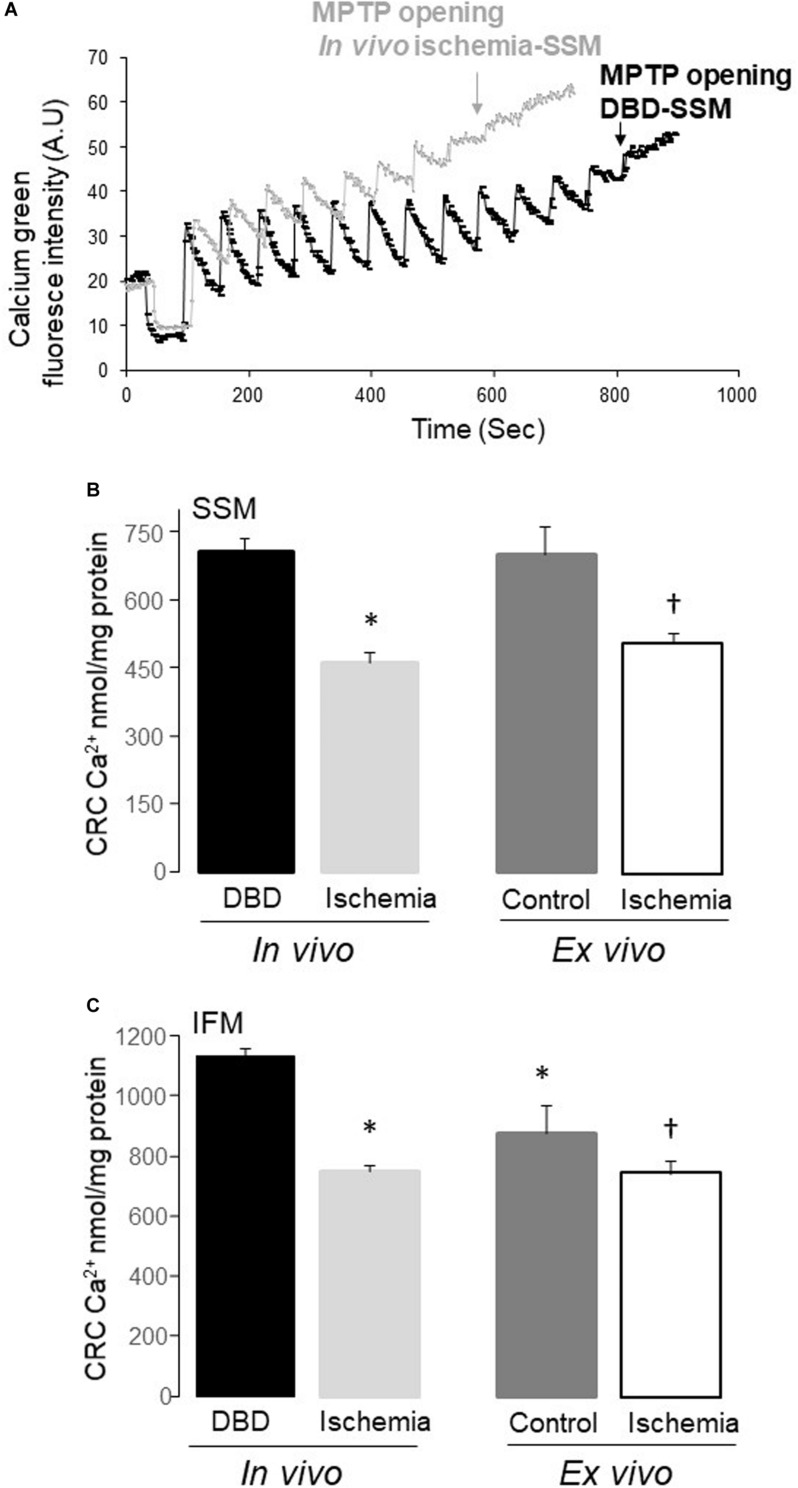
The mitochondrial permeability transition pore (MPTP) opening was increased in both subsarcolemmal mitochondria (SSM) and interfibrillar mitochondria (IFM) following *in vivo* and *ex vivo* ischemia. The calcium retention capacity (CRC) was used to reflect the MPTP opening. Original tracing of the CRC measurement was shown in **(A)**. The pulses of calcium given at one-minute intervals to induce MPTP opening in SSM from *in vivo* ischemia hearts were decreased compared to SSM from DBD hearts **(A)**. The CRC was decreased in both SSM and IFM from *in vivo* ischemia hearts compared to donation after brain death (DBD) hearts **(B,C)**. The CRC was also decreased in SSM and IFM from *ex vivo* ischemic hearts compared to time control hearts **(B,C)**. Interestingly, the CRC was relatively lower in IFM from time control hearts compared to DBD hearts **(C)**, indicating that buffer-perfusion alone may sensitize to MPTP opening. A.U, arbitrary units. Data are expressed as mean ± SEM; *p < 0.05 vs DBD; ^†^*p* < 0.05 vs. time control. Number of rat hearts for each group are, DBD = 6, *in vivo* ischemia = 5, time control = 11, and *ex vivo* ischemia = 9. Statistical method: one-way ANOVA using the Student–Newman–Keuls analysis for groups comparisons with an individual comparison *p* < 0.05 considered significant.

The CRC in both SSM and IFM from *in vivo* ischemia hearts was decreased compared to DBD hearts, indicating an increased susceptibility to MPTP opening in *in vivo* ischemia heart mitochondria (Figures 3A,B). The CRC was also decreased in SSM following *ex vivo* ischemia (Figure 3A), whereas *ex vivo* ischemia did not significantly alter the CRC in IFM compared to time control (Figure 3B). Interestingly, the CRC in IFM from time control hearts was lower than that in IFM from DBD hearts (Figure 3B).

### *In vivo* Ischemia did Not Significantly Alter H_2_O_2_ Generation

The amount of H_2_O_2_ generation in SSM and IFM following *in vivo* ischemia using glutamate as complex I substrate was not significantly increased compared to DBD hearts [mean ± SEM, SSM-DBD (53.3 ± 2.9 nmol/min/mg) vs SSM-*in vivo* ischemia (50.7 ± 1.9), *p* = NS; IFM-DBD (56.6 ± 2.9) vs IFM-*in vivo* ischemia (51.2 ± 2.0), *p* = NS].

## Discussion

Heart transplantation is limited by the available donor hearts, and DCD hearts have the potential to expand the donor pool. There are several steps involved in clinical DCD heart transplantation to ensure a successful outcome. Our study is focused primarily on identifying and quantifying the damage to mitochondria in the DCD set-up. It is essential to develop a reliable and reproducible animal model to study the effects of warm ischemia in a DCD heart to propose potential interventions to mitigate the ill effects of IR injury, which are inherent to the DCD process. Our study quantified the differences that exist between the *in vivo* ischemia model based on current clinical DCD practice and the time-tested *ex vivo* ischemia model where most of the published data on ischemia and reperfusion emanated from. The main findings of our study are, (a) both *ex vivo* and *in vivo* ischemia damages OXPHOS function of SSM and IFM, (b) the proportion of damage to SSM and IFM including proton leak through the inner membrane are significantly higher with *ex vivo* ischemia, (c) this excess damage to mitochondrial ETC from *ex vivo* ischemia was not apparent when DNP was used to exclude the complex V from ETC, (d) time control hearts showed a decrease in SSM function compared to DBD hearts driven by greater state 4 respiration; and lastly, (e) the CRC was decreased in SSM and IFM with *ex vivo* and *in vivo* ischemia.

In developing the rat heart *in vivo* ischemia model, we carefully examined the correlation between the *in vivo* ischemia times with derangements in mitochondria (OXPHOS) and heart function quantified through direct measurement of left ventricle developed pressure (LVDP). With a shorter duration of ischemia, the myocardium suffered less damage. With increasing duration of *in vivo* ischemia hearts sustained greater damage, in these hearts, when the initial LVDP was <50% compared to control hearts, they did not show any signs of functional recovery upon reanimation over 60 min. However, when the residual heart function was >50% of control hearts, there was a trend toward slow but steady improvement in LVDP over 60 min of reanimation (Chen et al., 2019). The *in vivo* ischemia time where residual LVDP in the heart was >50% compared to the control heart was 25 min. At this, *in vivo* ischemia time of 25 min, the residual mitochondrial OXPHOS function was 60% compared to control hearts. Based on these observations, we selected 25 min of ischemia time as the maximal duration of ischemia from which the heart has the potential to recover function. In a study by Wyss et al. (2019), a correlation was sought between the varying warm ischemia times (21, 24, 27, 30, and 33 min) and myocardial/mitochondrial function in an *ex vivo* ischemia rat heart model. They noticed that *ex vivo* ischemia time of 27 min or more correlated with a significant decline in myocardial and mitochondrial function, including an increase in cytochrome *c* release (Wyss et al., 2019). While their model does not mirror our *in vivo* ischemia model, it supports our selection of 25 min of ischemia time for the present study. To our knowledge, there is no published literature corroborating *in vivo* and *ex vivo* ischemic damage to mitochondria that we described here.

In the present study, 25 min of *in vivo* ischemia led to decreased OXPHOS primarily in SSM, and to a lesser amount in IFM compared to DBD group. This finding supports the greater sensitivity of SSM to ischemic injury (Lesnefsky et al., 1997, 2017). However, the *ex vivo* ischemia decreased OXPHOS relatively equally in both SSM and IFM. These findings are in line with the previously published data from the *ex vivo* ischemic rat heart mitochondria (Chen et al., 2006a). More importantly, when we compared the relative damages to mitochondria between *in vivo* and *ex vivo* ischemia groups, we noticed a substantially higher proportion of damage in *ex vivo* ischemia group (Tables 1, 2). It is reasonable to state that *in vivo* ischemia causes relatively less damage to mitochondria in particular to IFM compared to *ex vivo* ischemia. Since IFM are the primary source of energy supply to the contractile apparatus (Lesnefsky et al., 2016, 2017), the *ex vivo* ischemia likely decreases residual heart function proportionately more compared to *in vivo* ischemia for the same duration of ischemia. These findings need to be studied further in a working heart model and should be kept in perspective when evaluating interventions designed to improve heart function following global ischemia.

Our previous work and that of others have demonstrated that a decrease in respiratory enzyme activity contributes to decreased OXPHOS in mitochondria following *ex vivo* ischemia (Lemasters and Nieminen, 1997; Lesnefsky et al., 2001a; Chen et al., 2008). Therefore, we examined the enzyme activities in SSM and IFM from *in vivo* ischemia hearts to localize the defect in mitochondria contributing to decreasing in OXHOPS. We used the CS assay in mitochondria from DBD hearts and *in vivo* ischemia hearts to control for comparable mitochondrial extraction. The CS activity was comparable in mitochondria from DBD and *in vivo* ischemia groups. Our previous work has shown that the activity of complex IV is relatively preserved both in SSM and IFM even following prolonged (45 min) *ex vivo* ischemia (Lesnefsky et al., 1997), we therefore focused in the present study on examining complexes II, III, and cytochrome content. Cytochrome *c* plays a key role in transferring an electron from *c*1 to cytochrome oxidase (Lesnefsky et al., 2001b). Our study shows that the enzyme activities of complex II and III were not significantly changed in mitochondria from *in vivo* ischemia hearts compared to DBD hearts. Complex III is a centerpiece, which shuttles electrons from complex I to cytochrome *c* and complex IV (Coligan et al., 2000; Lesnefsky et al., 2001a). Glutamate is an NADH-linked complex I substrate with electron flow from complexes I to III, cytochrome *c* and into complex IV. A decrease in glutamate oxidation can be due to decreased activities of complex I, III, loss of cytochrome *c*, or a decreased cytochrome oxidase activity. In our study, the enzyme activity of complex III is not decreased in SSM or IFM from *in vivo* ischemia hearts, but the cytochrome *c* and aa3 contents were reduced (Tables 3, 4). These results support that loss of cytochrome *c* content contributed to decreased glutamate oxidation in *in vivo* ischemia mitochondria, although we cannot exclude a complex I defect entirely. The loss of cytochrome *c* from mitochondria leading to decreased OXPHOS was also noted in previous *ex vivo* ischemia studies (Lesnefsky et al., 1997; Chen et al., 2006a). Succinate donates reducing equivalents to complex II with electron flow to III, *c*, and IV. Since activities of complex II and III are not decreased in SSM and IFM from *in vivo* ischemia hearts, the loss of cytochrome *c* and potential cytochrome oxidase defect likely have played a role in decreased succinate oxidation in *in vivo* ischemia mitochondria. TMPD donates electrons to cytochrome *c* and cytochrome oxidase (Lesnefsky et al., 1997). We noticed a decreased OXPHOS in SSM and IFM from both *in vivo* and *ex vivo* ischemia hearts when TMPD-ascorbate was supplied as a substrate. The proportionate decrease in OXPHOS between *in vivo* and *ex vivo* groups compared to their controls is comparable (Tables 1, 2). A loss of cytochrome *c* content from both *in vivo* and *ex vivo* groups would explain this finding. Similar findings were also reported in previous *ex vivo* ischemia studies (Lesnefsky et al., 1997). Myocardial ischemia not only impairs the mitochondrial ETC but also inhibits metabolic enzymes, including pyruvate dehydrogenase (Thompson et al., 2016; Chen et al., 2018) and glutamate dehydrogenase (Lucas and Szweda, 1999). These metabolic enzyme activities may also be decreased in mitochondria from *in vivo* ischemia hearts, but were not studied here.

Another finding that was common to both forms of ischemia in our study was mitochondrial CRC. CRC was significantly impaired in both forms of ischemia and to a comparable level (Figure 3). MPTP opening is known to be inhibited during ischemia due to intracellular acidification (Xu et al., 2014) and the accumulation of ADP (Weiss et al., 2003). In the current study, although hearts only underwent ischemia without reperfusion, the CRC was significantly decreased in both ischemia groups. It is important to recognize that MPTP protection may yield added benefits if instituted prior to the onset of ischemia.

When examining the differences between the extent of derangement in mitochondrial function between *ex vivo* and *in vivo* ischemia, it is clear that the magnitude of damage is greater with *ex vivo* ischemia. This was seen across the OXPHOS function parameters of both SSM and IFM (Tables 1, 2). These differences need to be reconciled in the light of pathophysiologic changes that are innate to the *in vivo* ischemia (DCD) process but are not seen with the *ex vivo* ischemia model. While the *ex vivo* model allows for an abrupt cessation of oxygen delivery in an isolated heart via non-blood based perfusate, the *in vivo* ischemia process is relatively gradual and occurs in the setting of residual blood. There are distinct hormonal and hemodynamic changes that occur with the brain and circulatory death process (Bittner et al., 1995; Perez Lopez et al., 2009). In the experimental models (Bittner et al., 1995; Perez Lopez et al., 2009) and in human organ donors (Perez Lopez et al., 2009) catecholamine levels were increased by up to eight times the baseline values following brain death. Interestingly, histologic changes characteristic of contraction band necrosis were noted in 62% of donor heart specimens (Perez Lopez et al., 2009). The physiologic implications of this surge are not widely studied and are not part of the *ex vivo* ischemia models.

With the growing interest in DCD organ transplantation, studies were undertaken to observe hormonal and hemodynamic changes in animal models. In a porcine model of DCD, White et al. (2016) noted an immediate pulmonary vasoconstriction and right ventricle distention following the withdrawal of ventilatory support. After a transient hyper-dynamic state, a steady decline of heart function with the total circulatory arrest was noted. More importantly, oxygen delivery (tissue hypoxia) decreased from a baseline of 212 mL O_2_/min to 41 mL O_2_/min in less than 1.5 min following the cessation of ventilation. A corresponding drop in systolic BP was also noted (71 to 54 mmHg) along with a drop in arterial PO_2_ from 107 to 23 mmHg. It is clear from this seminal study that in less than 2 min from the withdrawal of ventilation, effective tissue hypoxia sets in. By magnetic resonance imaging, the right ventricle size increased by 18% and left ventricle size decreased by 12% from baseline values. Since mitochondria are responsive to mechanosensitive stimuli (Bartolak-Suki et al., 2017), these dynamic changes in heart chambers would have implications for mitochondrial function in the *in vivo* ischemia model but not in the *ex vivo* ischemia model. More importantly, mitochondrial mechanotransduction plays a key role in the regulation of intracellular calcium (Kim et al., 2018). Ryanodine receptor 2 (RyR2) channels that play a key role in sarcoplasmic reticulum release of calcium with myocyte depolarization are dysfunctional in disease states such as heart failure and atrial fibrillation where the cardiac chambers are known to distend and stretch (Hove-Madsen et al., 2004; Fischer et al., 2013). Since the *ex vivo* ischemia model does not undergo similar chamber distention/mitochondrial distortion, the effect of these changes needs to be considered while interpreting or comparing results from *in vivo* ischemia model. To what extent these changes contributed to the better residual function of *in vivo ischemia* mitochondria compared to *ex vivo* ischemia mitochondria in our study is difficult to ascertain.

Also, the DCD model, as described by White et al. (2016) showed that following termination of ventilation oxygen levels in blood continued to be present for at least 20 min, albeit in small amounts compared to the baseline (from 107 to 13 mmHg). The continued presence of oxygen during the *in vivo* ischemia process would have complex implications for myocyte/mitochondria function. While the anaerobic state that developed secondary to hypoxia may have facilitated a decrease in ROS production (through the blockage of ETC) but the supply of small amounts of oxygen may also have promoted the production of ROS (Duranteau et al., 1998; Vanden Hoek et al., 1998). Similarly, metabolic acidosis was noted 1.5 min following termination of ventilation and persisted throughout the 20 minutes of monitoring. This should facilitate maintaining the closure of MPTPs (Xu et al., 2014). If cellular acidity favors MPTP integrity in *in vivo* ischemia model, the effect of it was not as pronounced in our study. The decreases in CRC with both forms of ischemia in our study were comparable (Figure 3).

Reactive oxygen species can be either detrimental or beneficial to a cell depending on the amount, duration, and location of production. In our study, the ROS production in SSM and IFM from *in vivo* ischemia heart was comparable to control DBD hearts. Mitochondria are the major source of ROS production in a cell (Chen et al., 2003). While ROS production has been studied only in the pathological states such as ischemia and reperfusion, ROS also have physiologic functions, the importance of which are being studied more recently (Lee et al., 2011; Scialo et al., 2017). Mitochondrial ROS plays a critical role in cell signaling. Complex I transforms from an active state (A) to a dormant state (D) during ischemia. The D state produces less ROS, but the signaling process for this transformation is ROS, itself (Stepanova et al., 2019). ROS production is closely linked to cellular oxygen levels, cellular differentiation, lifespan extension and ETC rearrangement (Lee et al., 2011; Guaras et al., 2016; Scialo et al., 2016, 2017). ROS also regulates the arrangement of ETC into supercomplexes leading to efficient respiratory activity as demanded by the cell (Acin-Perez and Enriquez, 2014). To what extent the ROS generation influences the cell bioenergetics and mitochondrial function in an *in vivo* ischemia model is not known but should be considered when interpreting results from an *ex vivo* ischemia model.

The presence of blood in contact with myocytes during *in vivo* ischemia may also have played a role in different levels of mitochondrial OXPHOS function derangements. Since blood is a natural buffer and remains in contact with myocytes as the *in vivo ischemia* process evolves, it is possible that the deleterious effects of ischemia to mitochondria in *in vivo* ischemia hearts are in part protected. It is interesting to note that in our study, none of the *in vivo* ischemia hearts went into ventricular fibrillations upon the termination of ventilation; however, it is common to note ventricular fibrillation as the ischemia progressed in the *ex vivo* model (data not shown). These findings were also corroborated in the porcine DCD model, where only three out of sixteen hearts developed ventricular tachycardia or ventricular fibrillation and the rest maintained an organized ventricular activity through the 20 min of observation. This is an intriguing finding as pig hearts are very prone to arrhythmias with both hypoxia and acidosis (Matsuura et al., 2017).

Lastly, our prior study demonstrated that with non-blood based perfusates, it is not possible to provide enough oxygen to a beating heart *ex vivo* to keep its metabolic condition in an aerobic state (Quader et al., 2018). This fact may explain the findings of decreased OXPHOS, especially in SSM of buffer-perfused hearts compared to DBD hearts (Tables 1, 2). Hence, the buffer-perfused heart is not necessarily similar to the DBD heart when it comes to assessing mitochondrial function.

It is reasonable to state that *in vivo* ischemia causes relatively less damage to mitochondria in particular to IFM compared to *ex vivo* ischemia. Since IFM are the main source of energy supply to cardiac contractile apparatus (Lesnefsky et al., 2016, 2017), the *ex vivo* ischemia likely decreases residual heart function proportionately more compared to *in vivo* ischemia for the same duration of ischemia. Similarly, with less damage to IFM with *in vivo* ischemia, they are less susceptible to reperfusion injury and perhaps better heart function recovery with interventions compared to *ex vivo* ischemia hearts. These findings are of significance in predicting the response to an intervention aimed at resuscitating *in vivo* ischemia hearts. In particular, a lack of desired response to an intervention with *ex vivo* model may still yield beneficial results with *in vivo ischemia* model.

**FIGURE 4:**
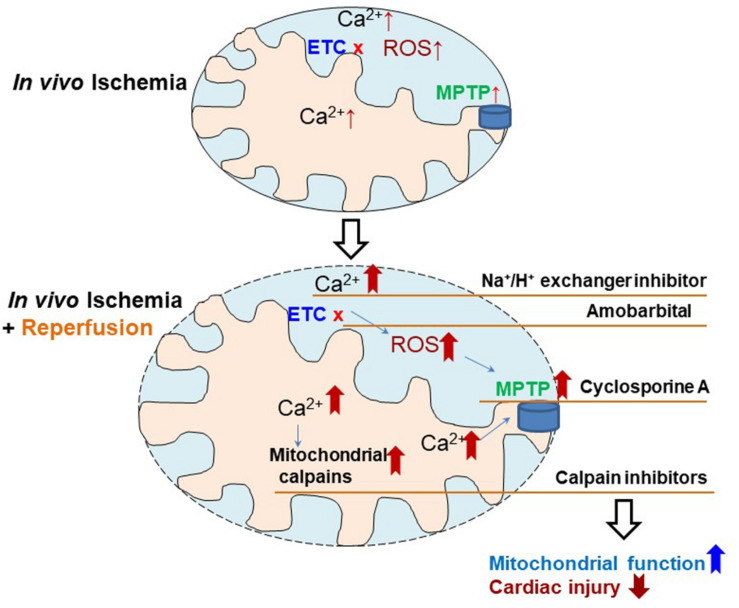
Depiction of the strategies to reduce cardiac injury in *in vivo ischemia* hearts. Ischemia mediated electron transport chain (ETC) damage contributes to cardiac injury, which is further exacerbated by reperfusion through, inducing intracellular calcium overload, increasing reactive oxygen species (ROS) generation, sensitizing mitochondrial permeability transition pore (MPTP) to opening, and activating mitochondrial calpains. Therefore, therapeutic strategies using Na^+^/H^+^ exchange inhibitor (decreasing calcium overload), amobarbital (reducing ROS generation), cyclosporine A (inhibitor of MPTP opening), and MDL-28170 (calpain inhibitor), may decrease cardiac injury in *in vivo ischemia* hearts during ischemia and reperfusion.

Our study identified the mitochondrial defect in *in vivo* ischemia hearts, which has been studied previously in *ex vivo* ischemia models. Based on these mitochondrial defects, the potential strategies to decrease injury in *in vivo* ischemia hearts include (a) using H^+^/Na^+^ exchanger inhibitors (Cropper et al., 2003) to decrease calcium overload, (b) amobarbital a reversible complex I ETC inhibitor, to decrease ROS generation especially at reperfusion (Xu et al., 2014), (c) cyclosporine A to stabilize MPTP and prevent them from opening (Halestrap et al., 2004), and (d) MDL-28170 (Thompson et al., 2016; Cao et al., 2019),an inhibitor of calpains to maintain calcium homeostasis (Figure 4). The effectiveness of these strategies need to be studied individually and in combination as the mitochondrial functions are quite integrated and interrelated.

## Limitations

A small sample size may not have the power to uncover differences that may further exist between the *in vivo* ischemia and *ex vivo* ischemia model. Reperfusion injury further augments the ischemic injury to mitochondria, which was not studied here. Contact with blood during *in vivo* ischemia process may have afforded protective mechanisms to mitochondria that are not accounted for in *ex vivo* ischemia model. Isolated mitochondria are commonly used to assess mitochondrial function in the hearts following ischemia-reperfusion. Since mechanical forces are required to permeabilize the cell membrane to release mitochondria, some physical damage to mitochondrial cannot be avoided. Ischemia may increase cell and mitochondrial fragility and potential damage. The decreased protein yield in mitochondria from ischemic hearts suggests the potential loss of mitochondria during the isolation process. Had those mitochondria remained in the analyzed samples, the differences between the study and control samples would have been more pronounced. Measurement of respiratory enzyme activities from whole heart homogenate may be an alternative approach to address this question in the future. The use of different substrates is an important tool to localize defects at the ETC. Glutamate, succinate, and TMPD-ascorbate are used in this study as complex I, II, and IV substrates, respectively. Duroquinol (DHQ) is a complex III substrate used in our previous study (Lesnefsky et al., 1997). Using DHQ will help to localize the potential defect at complex III. However, DHQ is not commercially available at present. Therefore, direct measurement of respiratory enzyme activity is used to detect potential defects at the ETC in the current study. We did not measure the blood oxygen levels in the *in vivo* ischemia group of rats; however, studies from previous DCD animal models confirmed the onset of tissue ischemia in less than 2 min from the withdrawal of ventilation. Lastly, ethical aspects of organ transplantation from DCD donors differ from that of the DBD donors and are not within the scope of this basic science study. An interested reader may find discussions on this subject in relevant publications (Truog et al., 2001; Steinbrook, 2007).

## Conclusion

Injury to mitochondria is greater with *ex vivo* ischemia compared to *in vivo* ischemia. In particular, the oxidative phosphorylation function of SSM are severely impaired with *ex vivo* ischemia compared to *in vivo* ischemia. CRC is equally decreased with both forms of ischemia, even without reperfusion. Possible reasons for the relatively less damage to mitochondria in the setting of *in vivo* ischemia could be due to gradual onset of ischemia, exposure to low concentrations of oxygen during ischemia, myocytes remaining in contact with blood, and a complex interplay between metabolic acidosis, myocyte stretch, and excess catecholamine levels.

## Disclosure

Dr. Toldo is PI of investigator-initiated studies from Olatec Therapeutics LLC and Kiniksa Pharmaceuticals.

## Data Availability Statement

The raw data supporting the conclusions of this article will be made available by the authors, without undue reservation, to any qualified researcher.

## Ethics Statement

The animal study was reviewed and approved by the Hunter Holmes McGuire Veterans Administration Hospital, Virginia Commonwealth University, and Institutional Animal Care and Use Committee.

## Author Contributions

MQ, OA, ST, EL, and QC participated in the research design. OA, QC, and RC conducted the experiments. MQ, OA, EL, QC, LK, and RC performed the data analysis. MQ, EL, OA, LK, and QC wrote and contributed to the writing of the manuscript. All authors contributed to the article and approved the submitted version.

## Conflict of Interest

The authors declare that the research was conducted in the absence of any commercial or financial relationships that could be construed as a potential conflict of interest.
